# Tips, Tricks, and Guides in Descemet Membrane Endothelial Keratoplasty Learning Curve

**DOI:** 10.1155/2021/1819454

**Published:** 2021-08-17

**Authors:** Davide Borroni, Carlos Rocha de Lossada, Mohit Parekh, Kunal Gadhvi, Chiara Bonzano, Vito Romano, Hannah J. Levis, Aris Tzamalis, Bernhard Steger, Miguel Rechichi, Marina Rodriguez-Calvo-de-Mora

**Affiliations:** ^1^International Center for Ocular Physiopathology, The Veneto Eye Bank Foundation, Venice, Italy; ^2^Department of Doctoral Studies, Riga Stradins University, Riga, Latvia; ^3^Department of Ophthalmology, University Hospital Virgen de Las Nieves, Granada 18014, Spain; ^4^Department of Ophthalmology, Ceuta Medical Center, Ceuta 51001, Spain; ^5^Institute of Ophthalmology, University College London, London, UK; ^6^Department of Ophthalmology, Royal Liverpool University Hospital, Liverpool, UK; ^7^Eye Clinic, DiNOGMI, University of Genoa and IRCCS San Martino Polyclinic Hospital, Genoa, Italy; ^8^Department of Eye and Vision Science, Institute of Life Course and Medical Sciences, University of Liverpool, Liverpool, UK; ^9^2nd Department of Ophthalmology, Aristotle University of Thessaloniki, Papageorgiou General Hospital, Thessaloniki, Greece; ^10^Department Ophthalmology, Medical University of Innsbruck, Innsbruck, Austria; ^11^Centro Polispecialistico Mediterraneo, Sellia Marina, Italy; ^12^Department of Ophthalmology, Regional University Hospital of Malaga, Malaga 29010, Spain; ^13^Department of Ophthalmology, Qvision (Vithas Almeria), Almería, Spain

## Abstract

Lamellar keratoplasty is fast becoming the most popular form of corneal transplantation. The adoption of Descemet membrane endothelial keratoplasty (DMEK) in the management of Fuchs endothelial dystrophy and pseudophakic bullous keratopathy is partly responsible for this shift in the paradigm of management of corneal pathology. The learning curve of DMEK, however, has been proven to be much steeper than previous endothelial keratoplasty procedures. To ease the procedure, experts have proposed multiple innovative techniques from tissue preparation to graft unfolding to aid the more novice surgeon. Here, we collate and share tips and tricks from our collective experiences to support the learning curve and outcomes in DMEK for both the novice and more experienced corneal transplant surgeons.

## 1. Introduction

The most common causes of endothelial failure are Fuchs endothelial dystrophy (FED) and pseudophakic bullous keratopathy (PBK) following intraocular surgery [1]. These remain a common indication for corneal transplantation, and in spite of developments in cataract surgery, we continue to see patients with these conditions warranting corneal transplantation in our clinics [2–4]. In modern times, endothelial keratoplasty (EK) has become the gold standard of care in the management of endothelial dysfunction in otherwise healthy eyes, replacing penetrating keratoplasty (PK) in the management of FED and PBK. EK delivers more predictable refractive outcomes and stronger structural integrity than PK without the protracted need for postoperative suture management [5–7]. Since its introduction by Melles et al. in 2006, Descemet membrane endothelial keratoplasty (DMEK) has increasingly gained in popularity with demonstratable benefits over other forms of EK [8]. DMEK involves only the transplantation of the Descemet membrane (DM) and endothelium in contrast to Descemet Stripping Automated Endothelial Keratoplasty (DSAEK) where the donor graft includes a variable portion of stroma [9–11]. This may confer the major benefit of DMEK which is a significantly lower risk of immunologic rejection compared to DSAEK [12, 13]. In the United Kingdom, data demonstrates the rising popularity of DMEK, which now represents 38.1% of EK compared to only 18.7% in 2015. Despite this, the difficulty in adopting this new technique means that DMEK remains less popular than DSAEK across the globe. The challenges posed by DMEK to the corneal transplant surgeon include a new method of graft preparation, delivery, unfolding, and increased incidence of postoperative graft detachment [14, 15]. Here we share tips, tricks, and our experience with the aim of making DMEK surgery simpler and safer.

## 2. Graft Preparation

The first step towards successful DMEK surgery is to master the donor tissue preparation technique [16]. Although many different techniques have been proposed for DMEK graft preparation, there is no consensus as to which is the optimum [17]. The most commonly used techniques include pneumatic dissection [18], stripping methods, and many more [19, 20]. The stripping methods have been the most widely adopted and we suggest starting learning DMEK with these techniques. We currently use 2 standard DMEK graft stripping preparation techniques, depending on our setting. In an eye bank setting, we use a double trephine technique. It involves the use of 2 punches, a mark on the graft, and multiple checks of endothelial cells during the procedure. A DMEK graft prepared in an eye bank setting reduces the surgeon's stress level due to possible failure in tissue preparation before surgery [21]. The second method is used in a theatre setting before the operation. It is quicker and it involves the use of a single trephine. For beginners, we suggest starting using DMEK tissue prepared in an eye bank setting and planning the first surgeries with the use of prestripped tissues [22].

### 2.1. In the Eye Bank: The Double Trephine Technique

**Figure 1:**
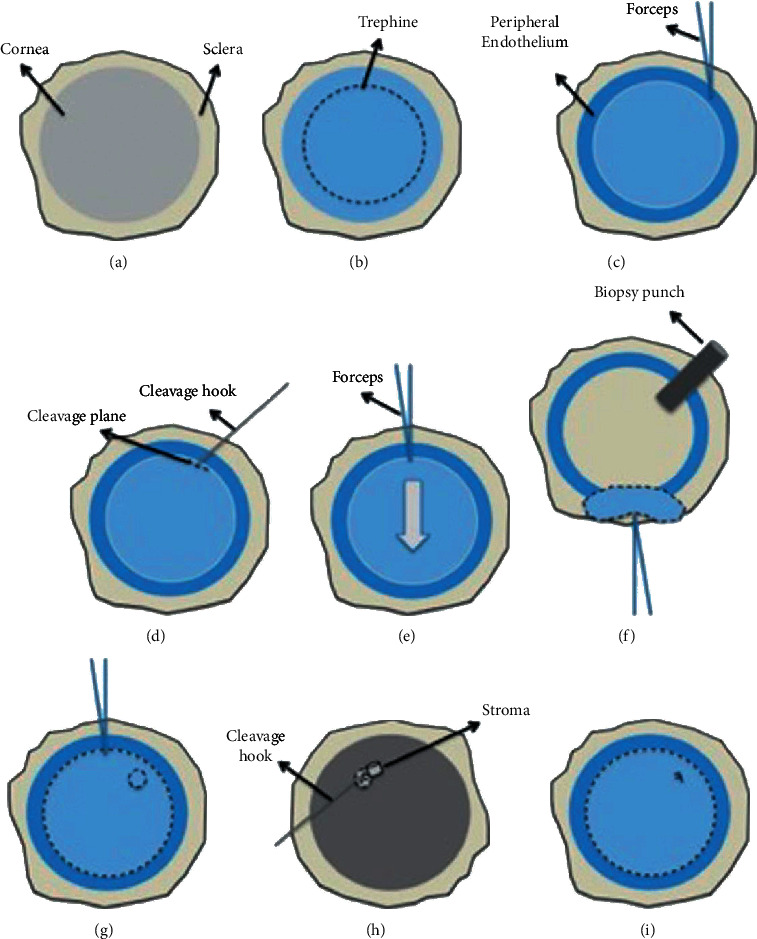
The double trephine technique: (a) healthy corneal tissue; (b) cut margins; (c) peripheral endothelium is removed; (d) separate the periphery of the central endothelium from the stroma; (e) the separated periphery is then grasped using the sharp acute forceps at the superior end and is peeled towards the inferior end; (f) a biopsy punch is used to create a small stromal punch; (g) the peeled DMEK tissue is replaced back on the stroma; (h) the tissue is marked; (i) endothelium is restained using trypan blue.

The corneal tissue is washed with sterile phosphate-buffered saline (PBS) to remove traces of storage media [23]. The cornea is then checked for endothelial cell mortality using trypan blue stain (0.025%) and endothelial cell density (ECD) is recorded using a calibrated graticule in the eyepiece of an inverted microscope. Average readings of 5 counts are usually obtained to avoid counting errors. If the tissue shows <5% trypan blue positive cells and >2200 cells/mm^2^, then it can be used for transplantation. The tissue is fixed on a vacuum block with the endothelium facing up (Figure 1(a)).

Using a corneal punch blade (9.5 mm), the endothelium is superficially trephined by gentle tapping on the top of the endothelium. Strong tapping or full thickness punches can end with the endothelium margins incarcerated in the corneal stroma increasing the preparation time. The cut margins are visualized using trypan blue stain (Figure 1(b)). The margin distinguishes the border between the central endothelium and the peripheral endothelium. Using sharp acute forceps, the peripheral endothelium is removed, leaving only the central endothelium (Figure 1(c)). To reduce radial tears and peripheral cuts of the tissue, we suggest using a cleavage hook to identify the cleavage plane and separate the periphery of the central endothelium from the stroma (Figure 1(d)).

The separated periphery is then grasped using the sharp acute forceps at the superior end and is peeled towards the inferior end (Figure 1(e)). The entire process may take a few to several minutes depending on the adherence of Descemet membrane (DM) to the underlying stroma. The tissue is peeled leaving approximately 10% of the inferior peripheral hinge. The hinge protects the DMEK tissue from free floating or forming a roll in the media. It is also helpful to allow stamping of the DMEK tissue on the DM side to avoid the tissue being transplanted upside down. Marking the tissue is not mandatory but it will ease DMEK unfolding. A biopsy punch is used to create a small stromal punch (Figure 1(f)) and the peeled DMEK tissue is replaced back on the stroma (Figure 1(g)). The vacuum is released, and the tissue is inverted on the vacuum block with the corneal epithelial side facing up. The punched stromal piece is then removed from the epithelial side. This allows gentian violet dye on the tip of a cleavage hook to be used to mark the letter “F” (with correct orientation) on the DM (Figure 1(h)). The stromal piece is returned and the tissue is inverted back and fixed on the vacuum holder. Although we have used letter “F,” other letters like “S” [24] can also be used. Once the tissue is ready, the endothelium is restained using trypan blue (Figure 1(i)) for final quality assurance of the graft in terms of ECD and mortality. The surgeon can then choose the diameter required for the patient and use a second trephine for excision of the graft before the transplant. We have observed minimal mortality and a high success rate using this technique [21]. Slight modifications such as oscillating movements, different points of initiation, and use of peripheral DMEK grafts have allowed us to manage challenging cases with tight adherence, cut/horse-shoe-shaped tears, and postcataract surgery tissues [23].

### 2.2. In the Operating Theatre: The Single Trephine Technique

**Figure 2:**
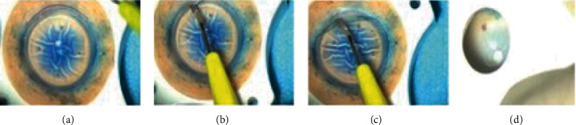
(a) Area selection; (b) beginning of peeling; (c) 100-degree 3 mm endothelium peeling; (d) tissue visualization.

This technique involves the use of only 1 trephine [23]. The cornea is centered on a punch base using suction. The vacuum is created with a syringe and the tissue is secured on the base. We start by staining the endothelium with trypan blue 0.06% (Vision Blue®; DORC, Zuidland, Netherlands) for 15–20 seconds. Thereafter, we identify an area in the periphery of the trabecular meshwork (TM) without damage, residual uveal tissue, or previous corneal incisions to start peeling the DM (Figure 2(a); marked in red).

DM is peeled from TM (Figure 2(b)) by gently swiping the DM layer from its periphery towards the center (Figure 2(c)) using a pediatric crescent knife of 2.3 mm, angled bevel up (Alcon Laboratories, Inc., Fort Worth, Texas). During this step, it is important to be careful not to apply too much pressure. If the crescent blade is too deep in the stroma, it will compromise the stripping by cutting into the stroma. The blade should be used perpendicularly to the cornea and an area of 100 degrees should be peeled for 3 mm towards the center of the cornea (Figure 2(c)). If this crucial step fails, it can be redone in another sector of the cornea leaving in place the managed endothelium.

The successfully peeled endothelium is then replaced back on the corneal stroma. The diameter of the punch is selected as required for the patient. The punch is placed on the graft and, before punching, the stripped and stained endothelium should be visible through the center of the punch (Figure 2(d)). If the trypan blue staining is not visible, the graft should be replaced in a different position or the peeled area should be increased. A donor cornea punch is then used to cut the graft. The size of the graft usually ranges from 8.25 to 9.5 mm in diameter.

Once the graft is cut, the cornea scleral rim is removed and the stripped area should be visible and possible to grasp. The forceps should not be pressed together too strongly. Higher grasping force could break the grasping point leading to a loss of tissue and need for regrasping potentially leading to higher loss of EC. The suction of the punch should be kept on throughout the procedure. If the surgeon has experience in DMEK stripping, a suction-free peeling could be considered. Having a mobile tissue to strip is more difficult to manage but it gives more freedom in the management of tensions and vectorial forces. The stripping is then completed with a longitudinal movement trying to avoid damages and tears [25].

During the peeling, high tension on the graft should be avoided to minimize the risk of ruptures. Sometimes however, stripping movement of the cornea could occur due to tension forces. In this case, additional toothed forceps could aid in keeping the corneal stroma in position. In the first cases, fast peelings are discouraged because they can create tight grafts [26]. Slow peeled grafts have the potential to ease unfolding during the DMEK surgery [26].

Once fully stripped, the tissue is placed on the corneal stroma and drops of preservative medium are placed on top of it. At this point, the patient should be called to theatre to start the surgery.

### 2.3. Graft Size

Graft diameter can vary as the size of the defective area changes. Corneal endothelial cell density (ECD) is higher in the periphery compared to the central cornea, especially beyond 9.00 mm [27, 28]. Delivering larger grafts could theoretically not only provide a higher number of transplanted cells but also include an area containing cells with high proliferative potential, which could potentially increase graft survival [29–31].

Although the use of a large DMEK graft is desirable in order to deliver more endothelial cells, the size of the graft must be carefully customized by measuring the white-to-white distance in cases that are not straightforward, such as Asian populations, high hyperopic eyes, and narrow anterior chambers, where smaller grafts are preferred. On the contrary, myopic and buphthalmic eyes can benefit from grafts larger than 9.5 mm [32]. In our experience, graft unfolding is more difficult when using DMEK grafts larger than 9 mm. We suggest that inexperienced surgeons who are new to the procedure should undertake their first cases using smaller graft diameters.

## 3. Preoperative Assessment: Anesthesia and Dilating Drops

We suggest performing DMEK surgery under topical anesthesia (TA) using Minims Proxymetacaine hydrochloride 0.5% w/v eye drop solution (Bausch & Lomb House, Surrey, UK) combined with peribulbar anesthesia (PA) with lidocaine 2% and bupivacaine 0.5% in a 3 : 2 ratio. We routinely use a 24-gauge needle and a trans-eyelid approach: the needle is inserted at a right angle to the skin at the lower orbital margin and advances 1.0–2.0 cm along the orbital floor at the temporal third of the lower eyelid with the eye in the neutral position of gaze, approximately 20 min before the surgery [33].

In cases where PA cannot be used, DMEK surgery can be safely performed under TA [34]. Indeed, the block can be avoided if surgery is brief, preferring TA with intracameral lidocaine [35]. Although levels of subjective pain are lower under PA than under TA, in pseudophakic patients without ocular comorbidities, Rickmann et al. suggest that TA combined with intracameral anesthesia could be considered, since it does not affect functional outcomes [36]. In agreement with them, in our opinion, it is feasible but it could complicate and prolong DMEK surgery for less experienced surgeons. Only experienced surgeons should use it for selected cooperative patients. Oral premedication with 15 mg midazolam or 10 mg diazepam before local anesthesia could be considered in anxious patients [37].

Sub-Tenon's capsule injection of local anesthesia is another method to achieve adequate local anesthesia for anterior segment surgery. Since any bleeding at the surgical site can track through the wounds and lead to fibrin formation in the anterior chamber (AC), the injection should be performed with caution to avoid large episcleral and conjunctival vessels.

**Figure 3:**
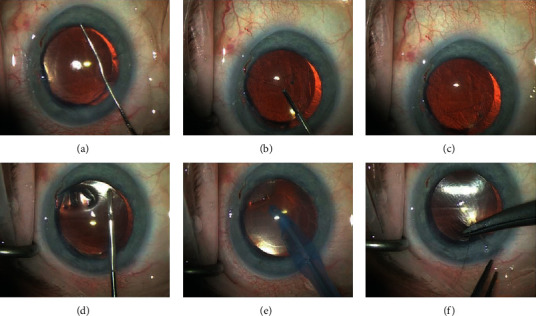
Descemetorhexis: (a) starting point; (b, c) peeling of the recipient Descemet's membrane and endothelium; (d) remnants removal; (e) AC washout; (f) suture.

Many surgeons perform DMEK with a peripheral iridotomy (PI) either prior to [38] or during the DMEK surgery to prevent air/gas bubble induced pupil block [39, 40]. However, an intraoperative PI is not without risks. Bleeding, glare, photophobia, lens capsule compromise, and vitreous strands through the PI are some of the complications reported to result from a surgical PI [41, 42]. We recommend a PI-less DMEK technique. This approach involves dilating the pupil with drops like tropicamide 1% or atropine 1% 30 minutes before surgery to obtain maximum dilatation. A dilated pupil helps to optimize red reflex, reduce the surface contact between iris and graft, and reduce the risk of pupillary block, and aids the visualization of the endothelium during descemetorhexis (Figure 3(a)). Conversely, we recommend a constricted pupil in selected cases like aphakic and vitrectomized eyes [43].

## 4. DMEK Surgery

### 4.1. Incisions

**Figure 4:**
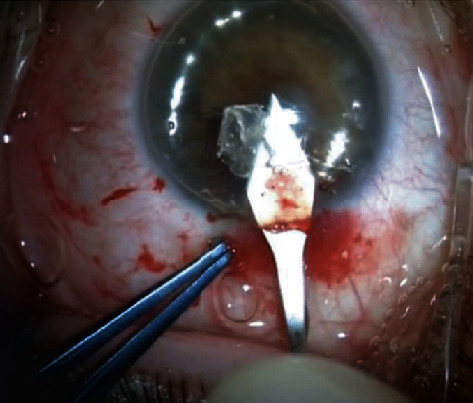
Main incision under air.

From early on, the DMEK technique has been carried out with small 3.0 mm, superior, 50% scleral depth, limbal, tunneled, self-sealing, sutureless main incision and three auxiliary paracenteses [8]. The technique for the main incision and auxiliary paracentesis has remained unchanged in the subsequent standardization of the technique [33]. Other publications describe the creation of a clear corneal main incision that ranges from 2.2 to 3.2 mm (Figure 4), depending on size and nature of the insertion device for the DMEK graft [39, 44]. We suggest placing the first 2 side ports 80–90° away from the main wound. They should be directed horizontally, so that the reversed Sinskey hook can be inserted avoiding the escape of the air placed in the AC and allowing the BSS to leave when shallowing the chamber. These ports will aid the unfolding process. The third side port should be more perpendicular as it will be useful at the end of the surgery to manage the level of air in the AC. The location and the placement of a suture on the main incision after graft insertion also varies greatly depending on the surgeon [44]. Nevertheless, DMEK can be considered a virtually sutureless procedure, increasing the postoperative refractive stability and decreasing the suture-related complications compared to previous keratoplasty techniques [45, 46].

### 4.2. Descemetorhexis

Descemetorhexis, the scoring and stripping of Descemet's membrane, is usually performed with a reversed Sinskey hook or a scraper or more rarely with a cystotome in a circular fashion. The usual diameter of descemetorhexis ranges from 8.5 to 9.5, depending on the size of the graft. The descemetorhexis can be performed under air or with the help of ophthalmic viscosurgical devices (OVD) which many will find easier when starting [47, 48]. Descemet's membrane visibility under air is superior, although the technique is technically more challenging due to air escaping during wound manipulation. Unless the AC is very shallow or there is posterior pressure, the need for air reinjection does not preclude the performance of descemetorhexis. If the surgeon needs more air, a continuous air infusion of a posterior vitrectomy device injection can be used by connecting an anterior chamber maintainer to the fluid air exchange system. Using a pars plana infusion can be useful in previously vitrectomized eyes.

Descemetorhexis under OVD has advantages such as a more stable anterior chamber, reduction of flare, and iris fluctuation [49]. If you choose this technique, we suggest performing at least a 180° descemetorhexis followed by the peeling of the recipient Descemet's membrane and endothelium with forceps (GRIESHABER® Asymmetrical Forceps, Alcon, Fort Worth, TX, US) (Figures 3(a)–3(c)). To facilitate the insertion of the forceps in the AC, we suggest bending it 60° in the middle. This is to avoid any contact/damage with the recipient corneal stroma. When required, forceps could aid with the refining and enlarging of the descemetorhexis reducing the donor-recipient overlaps (Figure 4(d)), in order to reduce the requirement for rebubbling.

It is important to completely remove OVD before inserting the graft, as it can interfere with graft adherence (Figure 3(e)). To double check if the AC is free from OVD, it is possible to insert a bubble of air and record its expansion. If OVD remnants are present, the air bubble will not expand in the AC. Additionally, air in the AC prevents swelling of the recipient cornea during the graft staining and loading phase.

At this point, a 10/0 nylon suture may be placed on the main incision. Performing this step before the insertion of the graft will facilitate faster suturing at the end of the surgery, avoiding major complications like expulsion of the graft from the AC or loss of air (Figure 3(f)).

### 4.3. DMEK Graft Staining, Loading, and Insertion

Staining the graft before its insertion into the AC is an important step for a successful and safe surgery. It is important to obtain a thoroughly stained graft, as it will aid with proper visualization during unfolding. The traditional dye used is 0.06% trypan blue (Vision Blue™, D.O.R.C. International) for 1-2 minutes [14], depending on the characteristics of the graft. Tight rolls may take longer to stain. The graft is then placed in a pot with BSS to facilitate the loading process. We suggest using a pot with a low height wall to ease the loading of the graft. To insert the prepared DMEK graft into the AC requires a specialized injector. Ideally, it should facilitate the loading of the graft, cause minimal cell loss/damage to the endothelium, and preserve the AC volume upon insertion. Surgeons may choose from a range of different insertion devices available commercially [50]. In our experience, we found that it was easier to start with glass injectors such as the Geuder glass tube (Geuder AG, Heidelberg, Germany). During the loading of the graft, the injector should be full of BSS and its tip completely submerged under the BSS to avoid air being taken up. If the graft is loaded but air is present in the injector, we suggest trying to remove as much as possible. If air is accidentally injected in the AC, it could complicate the unfolding process and it should be removed. Prior to injection, the graft is key to recheck the orientation (it must look like a “double roll” with the hinge down and the flanges up). When injecting the graft, it is important to have a low AC pressure and flat AC. This is because when injecting the graft, BSS is also being injected and an elevated AC pressure could result in a torpedo reaction that will push the graft back outside the AC.

### 4.4. DMEK Unfolding and Air Injection

Graft unfolding is the most variable step in DMEK surgery. DMEK graft, when peeled and submerged in BSS, will spontaneously roll outwards, exposing the endothelium. This requires unfolding manipulation once the tissue is injected into the AC. At the end of the surgery, DM should be well attached to the stroma. If DM spontaneously rolls inwards, complete unrolling is required to allow it to roll in the correct manner, before it can be attached using a standard AC air or sulfur hexafluoride (SF6) gas as tamponade [49]. Patient selection is a critical step, as a number of patient factors greatly influence the surgical course. Both very deep and very shallow AC configurations can be a major challenge [51]. Tissue from older donors tends to form wider graft rolls, which consequently require less manipulation during surgery, and where possible they should be considered for complex surgery and recipient eyes with deep AC anatomy [52].

The presence of coexisting ocular pathologies, such as glaucoma tubes, anterior synechiae, iris malformations, and anterior chamber intraocular lenses, increases the risk of intra- and postoperative complications including AC bleeding, bubble dislocation into the vitreous cavity, and graft detachment. When first learning the DMEK procedure, such cases with higher complication risks should be avoided [53, 54]. An AC free from OVD, air, and fibrin remnants is the first prerequisite for a successful and safe unfolding.

**Figure 5:**
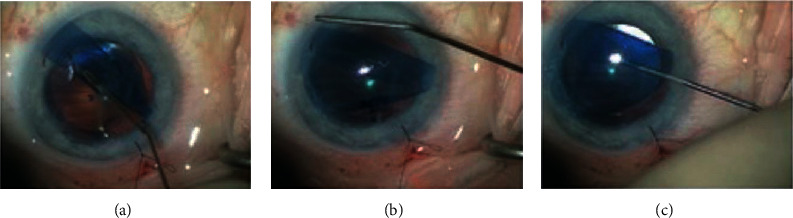
(a) Tap to unfold DMEK tissue; (b) tap to center the tissue; (c) DMEK orientation checks.

The surgeon must take care not to dislocate the DMEK graft in the vitreous chamber [55]. In postvitrectomy eyes, a temporary hydrophilic methacrylate sheet can be useful [56]. Different techniques to unfold DMEK grafts are reported in the literature [57–59]. For beginners, we suggest the tap technique: after the insertion of the graft, a suture to the main wound is closed and bordered. Short taps with 2 cannulas on the corneal surface and delicate bursts of BSS from the side ports help to open the graft and position it in the correct orientation (Figures 5(a)–5(c)). Fluid waves within the AC from the side ports as a result of corneal tapping also help to open the graft. In cases of a very tight scroll, an air bubble injected inside the scroll's lumen using a 30G-cannula may enlarge the scroll and help it to unfold [60].

It is better to keep the AC shallow but not completely flat [61]. For more experienced surgeons and as an approach to reduce the degree of graft manipulation in the AC, the endothelium can be manually tri-folded (taco-fold) endotethelium-in way, thus protecting the now inward endothelial cells and leaving the DM exposed. Tri-folded endothelium inward DMEK surgery is associated with similar endothelial cell loss compared to the endothelium-outward technique. Additionally, the mostly spontaneous unfolding of the graft inside the recipient eye reduces time and extent of surgical manipulation [62].

The correct orientation of the graft must be repeatedly verified during surgery and especially before attaching the graft to the posterior corneal stroma. The direct observation of the Moutsouris sign or the F mark is usually quick and helpful [59]. When the orientation is correct, a partly unfolded graft can be completely opened. If the graft is upside down, the anterior chamber can be deepened and a BSS burst between the iris and the graft will invert the graft in the AC.

Caution is advised when adding fluid to the AC during the unfolding process, as increased AC pressure may cause the graft to be flushed out of a corneal incision when entering the AC with an instrument. Even just minimal graft dislocation into a corneal wound will make surgery more complex and will result in endothelial cell loss in the affected graft areas. We therefore suggest placing a tight suture on the main incision and only then proceed to increasing AC pressure using BSS via the side ports. When the tissue is completely unscrolled and centered, air can be inserted under it to attach the graft to the recipient stroma [39, 63].

After air is injected in the anterior chamber, the centration of the graft can still be gently corrected using forceps (GRIESHABER® Asymmetrical Forceps, Alcon, Fort Worth, TX, US) to pull the graft into the desired position. To increase graft mobility, we suggest performing this procedure with no more than a 50% AC air fill. Despite the fact that this procedure may result in a small loss of endothelial cells, it will improve the centration of the graft. Improved centration again will result in faster corneal clearing and a lesser risk of peripheral graft overlap with recipient endothelium, thus reducing the likelihood of graft detachment [64].

At the end of the surgery, the vertical incision can be used to fill the AC with air, aiming to create a 90% air or gas fill. If available, intraoperative Optical Coherence Tomography (OCT) may facilitate all surgical steps by increasing the visualization of the graft and its orientation [65]. If the graft, despite rigid supine position of the recipient head during the early postoperative hours or days, detaches from the posterior corneal stroma and the detachment involves the pupil area or is seen to progress towards the pupil area, it needs to be reattached [66].

## 5. Conclusions

Tips and tricks can help surgeons new to DMEK to improve their outcomes and facilitate the uptake of DMEK surgery. A well-prepared DMEK graft and different surgical techniques improve the desired surgical outcome. The tips and tricks described in this article could be beneficial for new and experienced corneal surgeons.

## Data Availability

No data were used to support this study.
